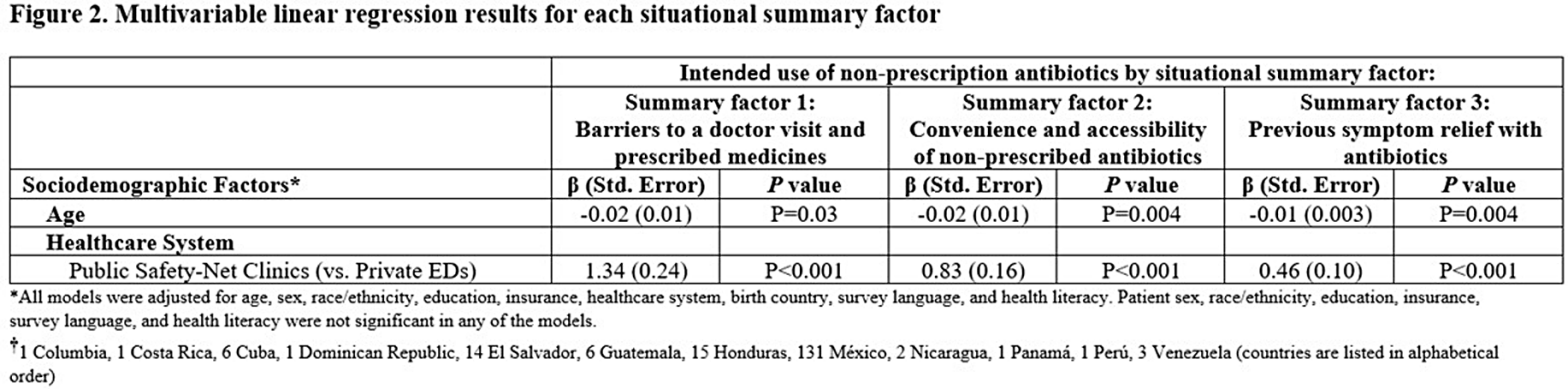# Situations Predisposing Primary Care Patients to Use Antibiotics Without a Prescription in the United States

**DOI:** 10.1017/ash.2024.124

**Published:** 2024-09-16

**Authors:** Lindsey Laytner, Barbara Trautner, Susan Nash, Fabrizia Faustinella, Roger Zoorob, Kiara Olmeda, Michael Paasche-Orlow, Larissa Grigoryan

**Affiliations:** Baylor College of Medicine, Department of Family and Community Medicine; Baylor College of Medicine

## Abstract

**Background:** Using antibiotics without medical guidance (non-prescription use) is a potential safety threat to individual and public health. Patients' situations can impact their intentions to use non-prescription antibiotics in the future (intended use). This survey (1) explores the dimensionality of 13 predefined situations to identify ‘summary factor,’ which include conceptually similar situations that influence patients’ intended use of non-prescription antibiotics, and (2) identifies the sociodemographic predictors associated with these summary factors. **Methods:** A cross-sectional survey was conducted from January 2020–June 2021 in the waiting rooms of six safety-net primary care clinics and two private emergency departments. We used principal component analysis as a data reduction technique and confirmed the factor structure of the situations (identifying three situational summary factors). Multivariate linear regression identified the sociodemographic predictors (e.g., age, gender, race, education, insurance, healthcare system, language preference, birth country, and health literacy) associated with each summary factor. **Results:** Of the 564 patients surveyed, the majority were female (72%), Hispanic or Latinx (47%), college-educated (44%), and received public health insurance (e.g., Medicaid or County Financial Assistance) (56%). The largest proportion of patients endorsed intended non-prescription antibiotic use for situations involving high doctor visit costs (29.8%), having leftover prescription antibiotics (50.4%), and experiencing symptom relief with prior use of antibiotics (47.5%) (Figure 1). We identified three situational summary factors: (1) perceived barriers to a doctor visit and receiving a prescription (Cronbach’s alpha [α]=0.96), (2) convenience and accessibility of non-prescription antibiotics (α=0.81), and (3) previous symptom relief with antibiotics (α=0.95). After controlling for gender, race, education, insurance, language preference, birth country, and health literacy, our multivariate regression results revealed that younger patients (P < 0 .04) and patients attending the safety-net health system (P < 0 .001) had more intended use of non-prescription antibiotics for all three summary factors (Figure 2). **Conclusions:** Our study revealed that younger patients and individuals receiving care from the safety-net clinics had an increased risk of intended non-prescription antibiotic use across all summary factors. Future stewardship interventions should consider the types of situations that drive patients' decisions to use antibiotics without a prescription. Interventions aimed at reducing barriers to healthcare (e.g., high costs and long waits associated with doctor appointments) and educating individuals on the risks associated with inappropriate antibiotic use while providing alternative (non-antibiotic) treatment options may reduce antibiotic use and antimicrobial resistance. **Acknowledgments:** This work is supported by grant number R01HS026901 from AHRQ and NRSA T-32 (6T32HC10031).

**Disclosure:** Barbara Trautner: Stock: Abbvie--sold in December 2023; Abbott Laboratories--sold in December 2023; -Bristol Myers Squibb--sold in December 2023; Pfizer--sold in December 2023; Consultant--Phiogen—consultant. Contracted research through NIAID for STRIVE trial, currently testing Shionogi product; Contracted research--Peptilogics; Contracted research--Genentech

**Figure:**
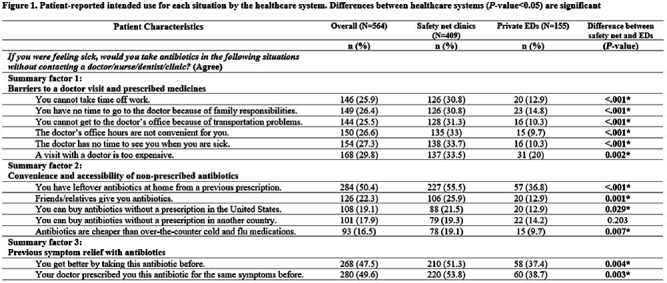

**Figure:**